# Renal mitochondrial restoration by gymnemic acid in gentamicin-mediated experimental nephrotoxicity: evidence from serum, kidney and histopathological alterations

**DOI:** 10.3389/fphar.2023.1218506

**Published:** 2023-07-13

**Authors:** Shubhangi Gumbar, Sudeep Bhardwaj, Sidharth Mehan, Zuber Khan, Acharan S. Narula, Reni Kalfin, Shams Tabrez, Torki A. Zughaibi, Samina Wasi

**Affiliations:** ^1^ Department of Pharmacology, Seth G. L. Bihani S. D. College of Technical Education, Institute of Pharmaceutical Sciences and Drug Research, Sri Ganganagar, Rajasthan, India; ^2^ Department of Pharmacology, ISF College of Pharmacy (An Autonomous College), Moga, Punjab, India; ^3^ Narula Research, LLC, Chapel Hill, NC, United States; ^4^ Institute of Neurobiology, Bulgarian Academy of Sciences, Sofia, Bulgaria; ^5^ Department of Healthcare, South-West University “NeofitRilski”, Blagoevgrad, Bulgaria; ^6^ King Fahd Medical Research Center, King Abdulaziz University, Jeddah, Saudi Arabia; ^7^ Department of Medical Laboratory Sciences, Faculty of Applied Medical Sciences, King Abdulaziz University, Jeddah, Saudi Arabia; ^8^ Department of Biochemistry, College of Medicine, Imam Abdulrahman Bin Faisal University, Alkhobar, Saudi Arabia

**Keywords:** nephrotoxicity, mitochondrial dysfunction, antioxidant, gymnemic acid, nephroprotection

## Abstract

**Background:** Nephrotoxicity refers to the toxigenic impact of compounds and medications on kidney function. There are a variety of drug formulations, and some medicines that may affect renal function in multiple ways via nephrotoxins production. Nephrotoxins are substances that are harmful to the kidneys.

**Purpose:** This investigation examines the renoprotective effect of gymnemic acid (GA) on Wistar rats in gentamicin-induced nephrotoxicity by analyzing serum, kidney, and histopathological markers.

**Study-design/methods:** The current study investigated the protective effect of GA at doses of 20, 40, and 60 mg/kg against gentamicin-induced nephrotoxicity in rats. Vitamin E was administered to compare the antioxidant capacity and efficacy of GA. In addition to the treatment groups, 100 mg/kg of gentamicin was administered intraperitoneal for 14 days. At the end of the study protocol, kidney homogenate, blood, and serum were evaluated biochemically. Serum creatinine, blood urea, glomerular filtration rate (GFR), mitochondrial dysfunctions, inflammatory cytokines, and renal oxidative stress were examined to assess gentamicin-induced nephrotoxicity. In addition, the impact of GA on the above-mentioned nephrotoxic markers were evaluated and further confirmed by histological analysis.

**Results:** This study establishes a correlation between antibiotic use, especifically aminoglycosides and acute renal failure. The research demonstrates the nephrotoxic effects of aminoglycosides, inducing mitochondrial ETC-complex dysfunction, and renal tissue inflammation in experimental rats. GA’s antioxidant properties restored renal oxidative stress markers, reducing kidney inflammation and injury. Histopathological analysis revealed a significant reduction in renal injury with GA treatment. Additionally, GA demonstrated greater efficacy than Vitamin E in restoring antioxidant potential and mitochondrial enzymes.

**Conclusion:** Consequently, our findings imply that long-term use of GA may be a suitable therapeutic strategy for reducing aminoglycoside toxicity. The current study suggests GA’s potential in treating gentamicin-induced nephrotoxicity and acute renal failure, meriting further investigation using advanced techniques.

## 1 Introduction

Nephrotoxicity is the harm done to the kidneys by outside and inside toxicants. This can cause problems with maintaining homeostasis and getting rid of toxins and waste (Al-Naimi et al., 2019). Long-term use of analgesics, antidepressants, antifungals, antibiotics, cardiovascular medications, chemotherapeutic agents, and other pharmaceuticals may have an adverse effect on the kidneys (Patel and Sapra, 2020). Approximately 20% of nephrotoxicity is caused by medications, while the remaining 80% depends on the individual’s lifestyle (Kim and Moon, 2012). Gold, interferons, NSAIDs, hydrazine, and lithium are nephrotoxicants that promote the progression of glomerulonephritis. Insoluble urine drugs, such as sulphonamide, trimethoprim, ampicillin, and acyclovir, cause crystal formation and tubular obstruction (Parazella, 1999; Hörl, 2010; Gianassi et al., 2019).

Aminoglycosides are frequently used due to their properties, which include rapid, concentration-dependent antibacterial effects, synergy with beta-lactam antibiotics, a low resistance rate, and low-cost therapy (Krause et al., 2016). There have been reports that gentamicin accumulation in the proximal renal convoluted tubule induces nephrotoxicity (Randjelovic et al., 2017). Nephrotoxicity is the most severe adverse effect of gentamicin, accounting for 10%–20% of all cases of acute renal failure (Mingeot-Leclercq and Tulkens, 1999). Patients’ serum creatinine levels rise within 2–44 days of drug exposure and earlier with subsequent re-exposure to the same drug (Griffin et al., 2019).

Medications that induce acute interstitial nephritis are believed to bind to antigens in the kidney or act as antigens then deposited in the interstitium, inducing an immune response. In gentamicin-induced nephrotoxicity, structural changes include cellular desquamation, glomerular atrophy, tubular necrosis, tubular fibrosis, epithelial edema of the proximal tubules, glomerular hypertrophy, perivascular edema, inflammation, and glomerular congestion (Schelling, 2016).

Tubular epithelial cells of experimental animals treated with gentamicin undergo apoptosis and necrosis. Cytotoxicity of gentamicin occurs in those cell types in which the antibiotic accumulates. These cells make up the epithelial cells of the cortex in the proximal tubule of experimental animals and humans in the kidneys (Randjelovic et al., 2017; Huang et al., 2020).

Inducing oxidative stress by increasing superoxide anions and hydroxyl radicals, cytosolic gentamicin contributes further to cell demise. Gentamicin causes mesangial contraction, decreasing UF (ultrafiltration coefficient) and GFR, and stimulates mesangial proliferation and apoptosis, which counteract one another (Pedraza-Chaverrí et al., 2003; Martínez-Salgado et al., 2004; Morales et al., 2006; Jiang et al., 2018). Gentamicin does not induce significant morphological changes in the glomerulus at large doses. Neutrophil infiltration has been associated with a modest increase in size, a change in round shape, and diffuse swelling of the filtration barrier (Lopez-Novoa et al., 2011).

Gentamicin directly increases the production of mitochondrial reactive oxygen species, which can damage numerous cellular molecules, such as proteins, lipids, and nucleic acids, impairing cell function and leading to cell mortality. Additionally, it contributes to mesangial and vascular contraction and inflammation, demonstrating that gentamicin’s nephrotoxicity involves an inflammatory response in both experimental animals and humans (Randjelovic et al., 2017).

Due to the nearly identical pharmacokinetic and toxicologic characteristics between rats and humans, rodents are a highly predictive human surrogate model for the nephrotoxicity of aminoglycosides. According to Mingeot-Leclercq and Tulkens (1999), the renal distribution and persistence of cortical concentrations of aminoglycosides in rats closely resemble those observed in humans.

The leaf of *G. sylvestre* has been extensively utilized in Ayurvedic medicine, where it is considered a bitter, acrid, thermogenic, digestive, liver tonic, analgesic, and anti-inflammatory. Due to the presence of triterpene saponins, known as, gymnemic acid (GA), gymnemasaponins, and gurmarin, the herb has an excellent inhibition property (Tiwari et al., 2014). Gymnemic acid is used to treat a variety of conditions, such as GIT disorders, ocular problems, as an antimicrobial agent, sweet bud repressing activities, kidney disorders, Hypercholesterolemia properties, allergies, asthma, and type 2 diabetes (Liu et al., 1992; Yoshikawa et al., 1992; Kanetkar et al., 2007).

In a study, it was found that GA had a positive effect on the kidney’s microvasculature and antiangiogenic properties that were associated with the expression of VEGF protein in the sectional and interlobar arteries of diabetic rodents given the diabetes-inducing agent (Komolkriengkrai et al., 2022). According to a study, the caffeic acid phenyethyl ester molecule (10 μmol/kg, i.p.) showed antioxidant and anti-inflammatory activity. The study’s findings showed decreased malondialdehyde (MDA) levels and increased superoxide dismutase (SOD), catalase (CAT), and reduced glutathione (GSH) activity. Thus, acting as a potent free radical scavenger to counteract the harmful effects of gentamicin (GEN) at biochemical and histological level (Parlakpinar et al., 2005). In another similar study, eugenol, a naturally occurring phenolic substance, was administered orally at a dose of 100 mg/kg before and after GEN treatment. It restored normal kidney function and reduced gentamicin-induced oxidative stress and hypoxia. The histopathological examinations further revealed its protective effect on GEN-induced renal tubular damage (Said, 2011). According to one more study, green tea extracts containing polyphenols, catechins, and their derivatives showed protective effects against GEN-induced nephrotoxic rats. This study observed that green tea extract at a dose of 300 mg/kg/day (taken orally for 7 days) prevented a further decrease in the levels of kidney function biomarkers such as GSH, SOD, and CAT, which were significantly reduced in gentamicin-treated rats (Abdel-Raheem et al., 2010). Similarly, the angiotensin-II receptor blocker drug, losartan at a dose of 10 mg/kg/day was found to be a renoprotective effect against GEN-induced kidney damage. In losartan + GEN-treated groups, the levels of MDA were reduced, while the levels of CAT and SOD were significantly improved compared to GEN-treated rats. Further histopathological examinations showed a protective effect on GEN-induced renal tubular damage (Heeba, 2011). The GA functioned by promoting anti-inflammatory, antioxidant, and enzyme activity, directly affecting kidney tissue (Tiwari et al., 2014; Jangam et al., 2023). In cases of diabetes mellitus, GA functions by stimulating the pancreas to produce more insulin. It similarly delays glucose absorption into the blood (Patel et al., 2009). Similarly, the angiotensin-II receptor blocker drug losartan at a dose of 10 mg/kg/day for 7 days found a renoprotective effect against GEN-induced kidney damage. In losartan + GEN-treated groups, the levels of MDA were reduced, while the levels of CAT and SOD were significantly improved compared to GEN-treated rats. Further histopathological examinations showed a protective effect on GEN-induced renal tubular damage (Heeba, 2011).

The present study aimed to examine the potential effects of GA on gentamicin-induced nephrotoxicity. The therapeutic effects were evaluated by determining body weight (g), estimation of serum creatinine, estimation of blood urea, estimation of protein in urine for assessment of nephrotoxicity, and estimation of kidney weight/body weight (%), in addition to a histopathological evaluation of renal hypertrophy and renal fibrosis. Comparing biomarkers such as urine volume, serum creatinine, and glomerular filtration rate between the treated and GA-treated groups reveals a significant difference in the current study. Vitamin E was also added to compare the antioxidant potential and efficacy of GA. Vitamin E has potential nephroprotective effects due to its antioxidative properties. Studies have shown that it reduces oxidative stress in kidneys, a leading cause of kidney diseases. In a study conducted on diabetic rats, vitamin E supplementation significantly improved kidney function and structure by reducing inflammation and oxidative stress (Jain and McVie, 1994; Kuhad and Chopra, 2009; Trujillo et al., 2013). Another research in patients with kidney stones demonstrated a positive correlation between vitamin E levels and kidney health (Singh et al., 2008).

Therefore, GA is beneficial in the ameliorative therapy of acute renal failure and can potentially counteract the oxidative stress caused by gentamicin. According to the conclusion, it was determined that GA at a dose of 60 mg/kg is an effective compound; therefore, additional research may be conducted using higher concentrations of GA.

## 2 Materials and methods

### 2.1 Experimental subjects

The experimental protocol employed received approval from the ‘Institutional Animal Ethics Committee’ under the guidelines given by the ‘Committee for Control and Supervision of Experiments on Animals (CPCSEA Reg No.**-**573/PO/Re/S/03/CPCSEA**)** ^1^Department of Pharmacology**,** Seth G. L. Bihani S. D. College of Technical Education Sri Ganganagar-335001 (Raj.). Wistar albino rats of either sex weighing about 200–300 g were used in the study. The animals acclimatized in the “Institutional animal house” and maintained on rat chow and tap water. They were kept under standard husbandry conditions of 12 h reverse light cycle with food and water *ad libitum*.

### 2.2 Drugs and chemicals

Gentamicin was obtained from the market (Genticyn; Abbott Healthcare Pvt., Ltd., Vitamin E capsules purchased from the market (Brand: Evion^®^ 200; Manufacturer: Merck limited). BAPEX, New Delhi, India, provide free samples of GA. All other chemicals utilized in the experiment are of analytical quality. Before use, solutions of the pharmaceuticals and chemicals are freshly prepared. The GA was water-soluble (Pathan and Bhandari, 2011).

### 2.3 Experimental protocol

**FIGURE 1:**
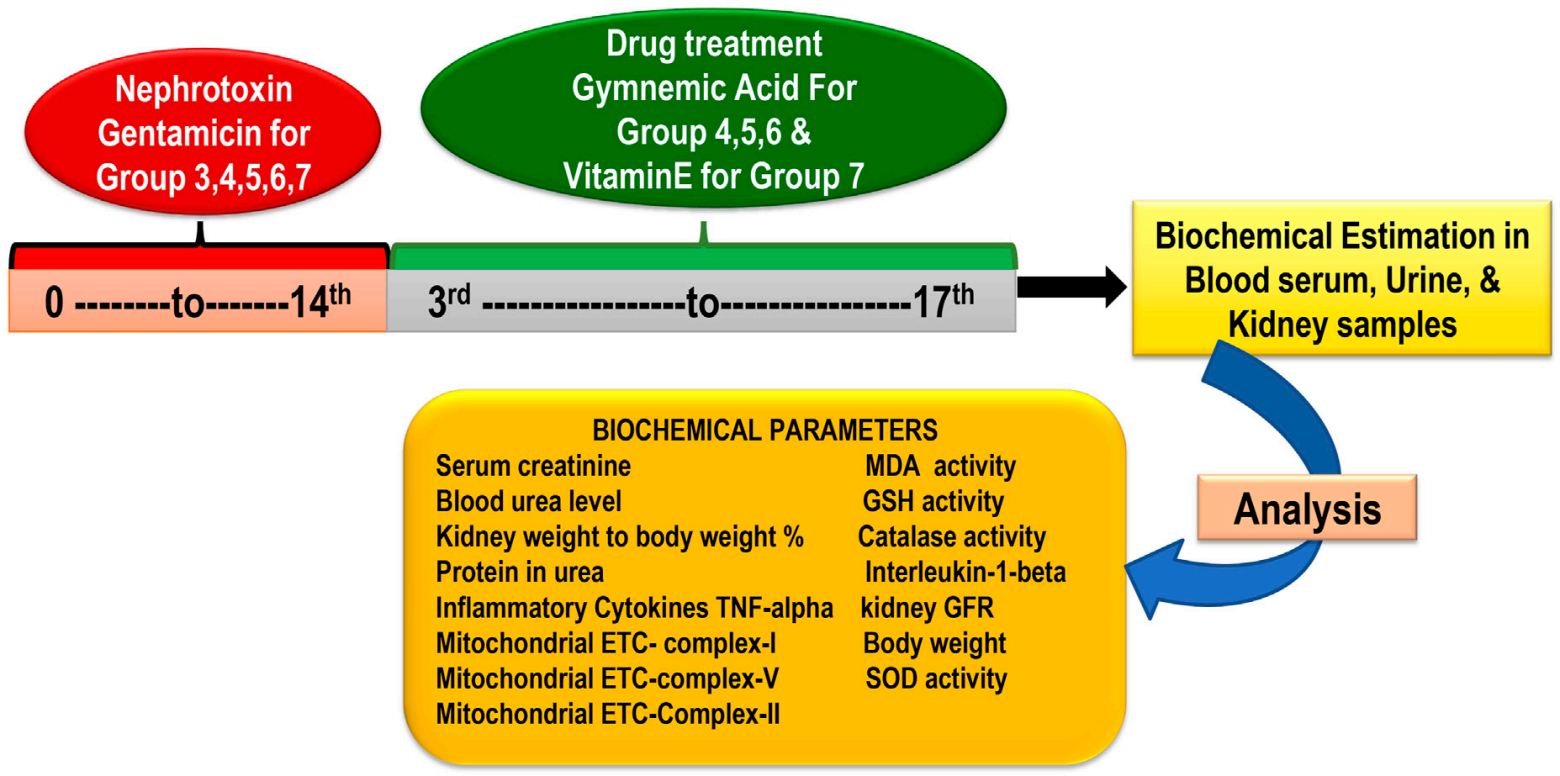
Experimental protocol schedule.

In the research, there are seven groups, each containing six rats. (Total rats: 42) Group 1 Control treated vehicle group (treated with water and standard food); Group 2 Gentamicin treated vehicle-treated (100 mg/kg/day, i.p.) for 14 days; Group 3 GA (60 mg/kg/day) (Orally for 14 days); Group 4 GA (20mg/kg/day,orally) + gentamicin (100 mg/kg/day, i.p.); Group 5 GA (40 mg/kg/day,orally) + gentamicin (100 mg/kg/day, i.p.), 2 weeks; Group 6 GA (60 mg/kg/day) + gentamicin (100 mg/kg/day, i.p.), 2 weeks; Group 7 Gentamicin + vitamin E (8 mg/kg/day, per os) orally for 14 days (Figure 1).

GEN administered intraperitoneally for 14 days at a 100 mg/kg dose induced nephrotoxicity in rodents (except control) (Udupa and Prakash, 2018). To dissolve GEN, normal saline was used. Based on a previous laboratory study, dosages of 20, 40, and 60 mg/kg of GA were selected (Daisy et al., 2009; Singh et al., 2016). The GEN dose (100 mg/kg) was determined based on the previous study’s findings (Udupa and Prakash, 2018). At the end of the study, the animal was euthanized with sodium pentobarbital (60 mg/kg, i.p.) and whole blood samples were collected from the retro-orbital plexus to obtain serum for renal function parameters (Pai et al., 2012; Sahu et al., 2014; Fahmy et al., 2017; Mohamed et al., 2020). Following the removal and rinsing of the kidney tissues in phosphate-buffered saline (PBS), the kidney weight/body weight (%) were recorded.

Rats’ retro-orbital plexus blood samples were collected using a semi-autoanalyzer into containers containing anticoagulant (2% sodium oxalate) to measure the concentration of blood serum urea and serum creatinine. One kidney was isolated using the lysate buffer for homogenization purposes and another for histological examination. The kidneys were quickly removed, washed with ice-cold normal saline, and homogenized in buffer solution (10%w/v). The homogenate was further used to estimate GSH, MDA, catalase, SOD, tumor necrosis factor-alpha (TNF-α), interleukin-1beta (IL-1β), and total protein. For histopathological analysis to gauge the severity of kidney damage, the remaining kidney tissue was fixed in 10% formalin (Figure 1).

### 2.4 Experimental gentamicin nephrotoxicity evaluation

#### 2.4.1 Estimation kits

Serum creatinine was estimated by the Jaffe-picrate method from a commercially available kit, ACM-Creatinine, marketed by: Acu Medica Lab Systems (P) Ltd., (Delanghe and Speeckaert, 2011). Blood urea is estimated by the urease method from a commercially available kit–UREA BEARTHLOT, marketed by: PRECISION BIOMED PVT., LTD. (Zawada et al., 2009). End-point assay for protein in urine using a commercially available kit (Autospan^®^ Liquid Gold Total Protein, marketed by ARKRAY Healthcare Pvt., Ltd., (Aitekenov et al., 2021).

#### 2.4.2 Estimation of serum creatinine

The serum creatinine concentration is estimated using Jaffe’s Method, Initial Rate, and commercially available reagent. Briefly, 100 µL of serum sample and 100 µL of standard creatinine solutions (2 mg/dL) are collected in specific test (T) and standard (S) glass tubes. In both containers, 1,000 µL of an alkaline picrate solution-containing working reagent is mixed, and the reaction temperature is maintained at 30°C. Spectrophotometrically, the absorbance of the test and standard at 20 s (T1, S1) and 80s (T2, S2) is measured against a blank. The formation of a colored complex owing to a reaction between creatinine in the serum sample and alkaline picrate in the working reagent is measured at 510 nm (Teslariu et al., 2016).

#### 2.4.3 Estimation of blood urea

The urease method estimates blood urea concentration using a commercially available test instrument. Briefly, 10 µL standard solutions (50 mg/dL) and 10 µL serum samples are each collected in different standard (S) and test (T) glass containers. The working enzyme reagent (1 mL) containing urease and hypochlorite is added and thoroughly mixed into each glass tube. All glass containers are incubated for 3 minutes at 37°C. Then, 1 mL of chromogen reagent is added, and the mixture is incubated at 37°C for 5 min to determine the absorbance of the test and standard at 578 nm relative to the blank. The underlying principle of this estimation is as follows. Urease converts urea to carbon dioxide and ammonia. The formed ammonia combines hypochlorite and phenolic chromogen to produce a green compound (Konieczna et al., 2012).

#### 2.4.4 Estimation of total renal protein urine

Using a commercially available kit (Coral clinical system, Goa, India), the total renal proteins were determined via the biuret method. To prepare the test, standard, and blank, 1,000 µL of biuret reagent was added to 50 µL of phosphate buffered saline 6% homogenate, 50 µL of standard albumin (8 g/dL), and 50 µL of purified water, respectively. After 10 min, the absorbances of the test and standard samples were measured spectrophotometrically at 540 nm against a blank (Zheng et al., 2017).

#### 2.4.5 Estimation of glomerular filtration rate (GFR)

The urine samples of each group were collected using the metabolic cage. GFR was calculated using the following formulas: GFR = UV/P, where U is the creatinine concentration in urine in mg/dl, V is the volume of urine produced per minute, and P is the plasma creatinine concentration in mg/dL. (Shahbaz and Gupta, 2019). The GFR rate of the control groups was compared with gentamicin–induced renal impairment (Udupa and Prakash, 2018).

#### 2.4.6 Estimation of kidney weight to body weight (%)

Separating the left and right kidneys, removing the renal fascia, and weighing each kidney separately. The kidney weight/body weight (%) is calculated using the formula:

$$Calculation=\frac{Left\;kidney\;weight\left(gm\right)+Right\;kidney\;weight\left(gm\right)X100}{Body\;weight\left(gm\right)}$$

The kidney weights of the various experimental groups were compared using the kidney weight per 100 g of body weight (Varatharajan et al., 2017).

#### 2.4.7 Evaluation of the body’s weight

The body weight of rats was assessed by a standard digital balance on the days 1st and 17th. To eliminate diurnal variations, the rats were generally weighed between 10:30 a.m. and 2.00 p.m.

### 2.5 Assessment of renal mitochondrial complexes and enzymes

In isolating rat kidneys for mitochondrial enzyme complex activities, the kidney was homogenized in an isolated buffer, and then homogenates were centrifuged at 13,000 × g for 5 min at 4°C. In isolation, buffer pellets were re-suspended with ethylene glycol tetraacetic acid (EGTA) and spun again at 13,000 × g at 4°C for 5 min. The resulting supernatants were transferred into the new tubes, and the isolation buffer was topped with EGTA and spun at 13,000×g at 4°C for 10 min. Without EGTA, pellets containing pure mitochondria were resuspended in an isolation buffer (Micakovic et al., 2019).

#### 2.5.1 Mitochondrial ETC complex-I enzyme estimation

The rate of NADH oxidation at 340 nm in an assay medium was used to spectrophotometrically evaluate the activity of complex-I at 37°C for 3 min. The rotenone-sensitive activity was observed in both the presence and absence of 2 µM rotenone (Mehan et al., 2017) and was complicated. The results were expressed in nM/mg units.

#### 2.5.2 Mitochondrial ETC complex-V enzyme estimation

To inactivate ATPases, aliquots of homogenates were rapidly sonicated in 0.1 N ice-cold perchloric acid. The ATP-containing supernatants were neutralized with 1 N NaOH and stored at 80°C until centrifugation (14,000 g, 4°C, 5 min) and subsequent analysis. PerkinElmer reverse-phase HPLC was used to measure the amount of ATP in the supernatants (Mehan et al., 2018). The reference solution of ATP was prepared according to the dissolving standard, and the wavelength used for detection was 254 nm. The results were expressed in nM/mg units.

#### 2.5.3 Mitochondrial ETC complex-II enzyme estimation

The absorbance at 490 nm was measured spectrophotometrically (Shimadzu, UV-1700) using the gradient fraction of homogenate with 0.3 mL of sodium succinate solution. The chromophore’s molar extinction coefficient (1.36 × 10^4^ M^1^ cm^1^) calculated results as INT reduced nM/mg protein (Kapoor and Mehan, 2021; Sharma et al., 2021).

### 2.6 Assessment of renal oxidative stress

The kidney was dissected and washed with ice-cold isotonic saline and weighed. The kidney was then minced, and a homogenate (10% w/v) was prepared in chilled 1.15% KCl. The homogenate was used to estimate GSH, MDA, catalase and SOD levels.

#### 2.6.1 Estimation of reduced glutathione (GSH)

The Ellman method was used to estimate the kidney’s GSH concentration. In brief, 10% w/v trichloroacetic acid was diluted 1:1 with the renal homogenate, and the mixture was centrifuged for 10 min at 5,000 rpm at 4°C. The resultant supernatant (0.5 mL) was combined with 0.4 mL of distilled water and 2 mL of a disodium hydrogen phosphate buffer with a concentration of 0.3 M. 0.25 mL of freshly synthesized 0.001 M DTNB [5, 5′-dithiobis (2-nitrobenzoic acid) blended in 1% w/v sodium citrate] was then added. After 10 min of incubation, the absorbance of the yellow complex was measured spectrophotometrically at 412 nm in the reaction mixture (Niknahad et al., 2017). The data were plotted on a standard curve using the reduced form of glutathione and reported in nmol/mg of protein.

#### 2.6.2 Estimation of malondialdehyde (MDA)

The quantitative measurement of MDA, a lipid peroxidation product–in kidney homogenate was performed as described earlier (Mehan et al., 2011). After the reaction with thiobarbituric acid, the amount of MDA was measured using a spectrophotometer at 532 nm. The MDA concentration was expressed as nM/mg of protein.

#### 2.6.3 Estimation of catalase enzyme

Renal homogenate catalase activity was determined by mixing 0.2 mL of tissue homogenate with 1.2 mL phosphate buffer (0.05 M, pH 7.0) and initiating the enzymatic reaction with 1.0 mL of hydrogen peroxide (0.03 M). The change in absorbance was measured at 240 nm for 3 min, while 1 mL of distilled water was used in place of hydrogen peroxide in the enzyme blank. The enzyme activity was computed based on the quantity of hydrogen peroxide oxidized/mg of protein (Sharma et al., 2021).

#### 2.6.4 Measurement of superoxide dismutase (SOD) levels

Using spectrophotometry, SOD activity was determined by auto-oxidation of epinephrine at pH 10.4. The supernatant of the kidney homogenate (0.2 mL) was mixed with 0.8 mL of 50 mM glycine buffer, pH 10.4, and the reaction was initiated with 0.02 mL epinephrine. After 5 min, the absorbance was spectrophotometrically measured at 480 nm. The SOD activity was quantified in µM/mg of protein (Sharma et al., 2019).

### 2.7 Assessment of renal inflammatory cytokines

#### 2.7.1 Measurement of TNF- α and IL-1β levels

Using a rat ELISA immunoassay kit (E-EL-R0019/TNF-α; E-EL-R0012/IL-1β; ELabSciences, Wuhan, Hubei, China). TNF-α levels were measured in rat kidney homogenate (Sharma et al., 2019) and blood plasma. The activity of IL-1β was measured in rat kidney homogenate and blood plasma as pg/mg protein (Tiwari et al., 2021).

### 2.8 Histopathological evaluation

The renal structural alterations induced by gentamicin were evaluated histologically by removing the kidney and immersing it in a 10% formalin solution. The kidney was then dehydrated in alcohol with increasing concentration, immersed in xylene, and embedded in paraffin. The paraffin blocks were sectioned to a thickness of 5 μm, stained with hematoxylin and eosin, and examined for pathological changes in glomeruli and tubules using a light microscope (Higher magnification views, 294*400) and a digital camera (Nikon, Japan). Histopathological investigations are conducted by Dr. Aditya Aggarwal of ADITYA PATH LAB in Sirsa, Haryana. Injected with gentamicin, the kidneys of rodents exhibited tubular, glomerular, and interstitial changes (Singh et al., 2018).

### 2.9 Statistical analysis

All results were statistically analyzed using Graph Pad Prism version 5.01 (Graph pad software, INC, La Jolla, CA, United States) and expressed as the mean ± standard deviation (SD). Results obtained from various groups were statistically analysed using one-way analysis of variance (ANOVA) followed by Post hoc Tukey’s test *p* < 0.05 was considered statistically significant.

## 3 Results

The present study aimed to investigate the possible effects of GA on gentamicin-induced nephrotoxicity. The therapeutic effects were evaluated using the difference in body weight (gm), estimation of serum creatinine, estimation of blood urea, estimation of protein in urine for assessment of nephrotoxicity. The estimation of kidney weight/body weight (%), and histopathological alteration study was performed for the assessment of renal hypertrophy and renal fibrosis.

### 3.1 Biochemical parameters

#### 3.1.1 Effect of GA on body weight in gentamicin-treated nephrotoxic rats

**FIGURE 2:**
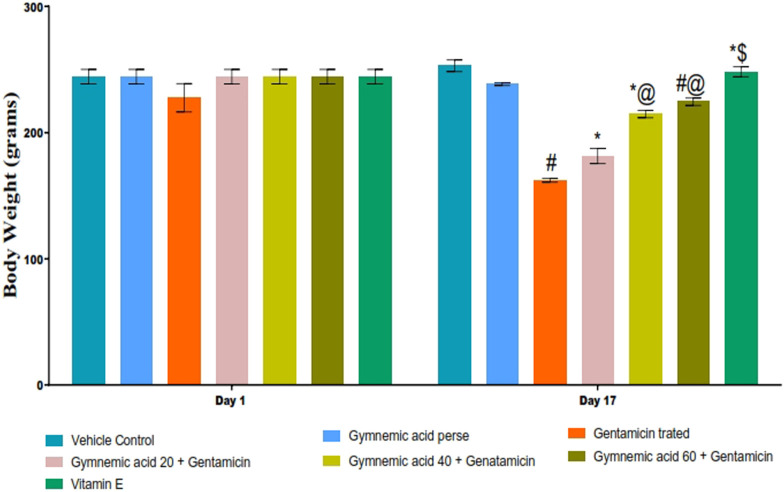
Effect of GA on body weight in gentamicin-treated nephrotoxic rats. Data depicting seven Groups per group of six rats in the above graph. Values were expressed as mean ± SD; Bonferroni, multiple comparison tests, analyzed data. ^#^ *p* < 0.001 versus vehicle control/GA 60; *p < 0.001 versus gentamicin treated; *@ *p* < 0.001 versus gentamicin and GA 20; #@ *p* < 0.001 versus gentamicin and GA 40; *$ *p* < 0.001 versus gentamicin and GA 60.

The body weight of rats treated with GEN was significantly (*p* < 0.01) lower than that of the control group. Compared to rats treated with GEN, oral administration of GA (20, 40, and 60 mg/kg, p.o., 2 weeks) significantly and dose-dependently restored body weight (*p* < 0.001) [F (6,35) = 97.07]. In addition, vitamin E (8 mg/kg) significantly (*p* < 0.001) increased body weight compared to GEN-treated rats after 17 days of observation (Figure 2).

#### 3.1.2 Effect of GA on GFR level in gentamicin-treated nephrotoxic rats

**FIGURE 3:**
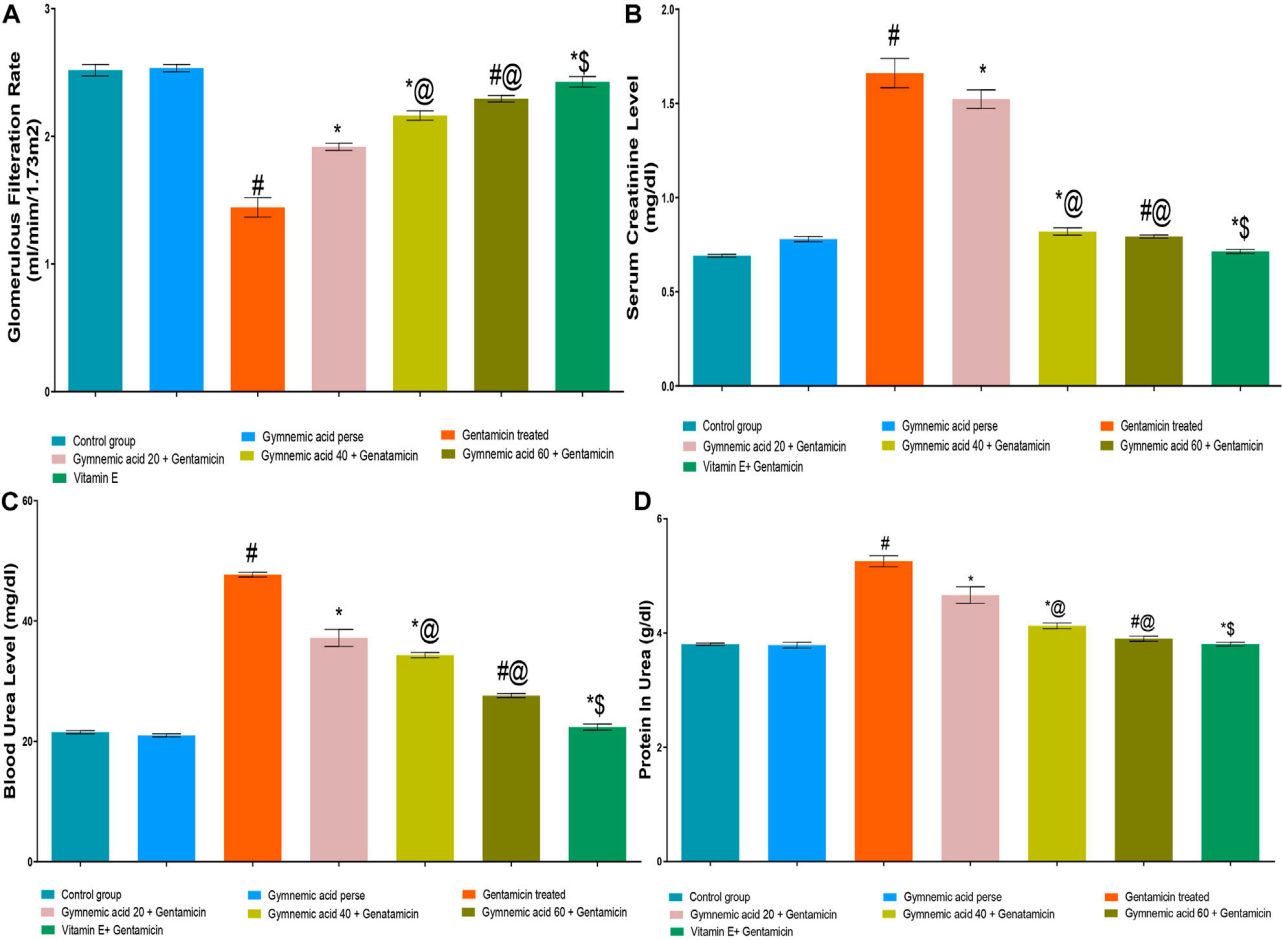
**(A–D)** Effect of GA on GFR **(A)**, serum creatinine **(B)**, blood urea **(C)** and protein in urea **(D)** in gentamicin-treated nephrotoxic rats. Data representing seven Groups per group of six rats in the above graph. Values were expressed as mean ± SD; Tukey’s multiple comparison tests analyzed data. ^#^ *p* < 0.001 versus vehicle control/GA 60; *p < 0.001 versus gentamicin treated; *@ *p* < 0.001 versus gentamicin and GA 20; #@ *p* < 0.001 versus gentamicin and GA 40; *$ *p* < 0.001 versus gentamicin and GA 60.

The GFR level of rats treated with GEN were significantly (*p* < 0.01) lower than those of the control group. GA (20, 40, and 60 mg/kg, p.o., 2 weeks) administered orally to GEN-treated rats significantly and dose-dependently increased the GFR rate (*p* < 0.001) in comparison to GEN-treated control rats [F (6, 35) = 491.9]. Moreover, vitamin E (8 mg/kg) increased GFR significantly (*p* < 0.001) compared to GEN control rats (Figure 3A).

#### 3.1.3 Effect of GA on serum creatinine level in gentamicin-treated nephrotoxic rats

The serum creatinine level of rats treated with GEN were significantly (*p* < 0.01) higher than those treated with a control group. GA (20, 40, and 60 mg/kg, p.o., 2 weeks) administered orally to GEN-treated rats significantly and dose-dependently decreased serum creatinine (*p* < 0.001) compared to GEN control rats [F (6, 35) = 766.5]. In addition, vitamin E (8 mg/kg) significantly (*p* < 0.001) decreased serum creatinine levels in rodents treated with GEN treated animals (Figure 3B).

#### 3.1.4 Effect of GA on blood urea level in gentamicin-treated nephrotoxic rats

GEN control rats had significantly (*p* < 0.01) higher blood urea levels than control rats. GA (20, 40, and 60 mg/kg, p.o., 2 weeks) administered orally to GEN-treated rats decreased blood urea significantly and dose-dependently (*p* < 0.001) compared to GEN-treated control rats [F (6, 35) = 1441]. In addition, vitamin E (8 mg/kg) decreased blood urea significantly (*p* < 0.001) compared to GEN-treated control rats (Figure 3C).

#### 3.1.5 Effect of GA on renal protein in urine in gentamicin-treated nephrotoxic rats

The protein level in the urine of GEN-treated control rodents was significantly (*p* < 0.01) higher than that of the control group. Oral administration of GA (20, 40, and 60 mg/kg, p.o., 2 weeks) to GEN-treated rats decreased urinary protein levels significantly and dose-dependently (*p* < 0.001) compared to GEN-treated control rats [F (6, 35) = 338.1]. Moreover, vitamin E (8 mg/kg) decreased urine protein levels significantly (*p* < 0.001) compared to GEN control rats (Figure 3D).

### 3.2 Kidney weight/body weight (%) in gentamicin-treated nephrotoxic rats

**FIGURE 4:**
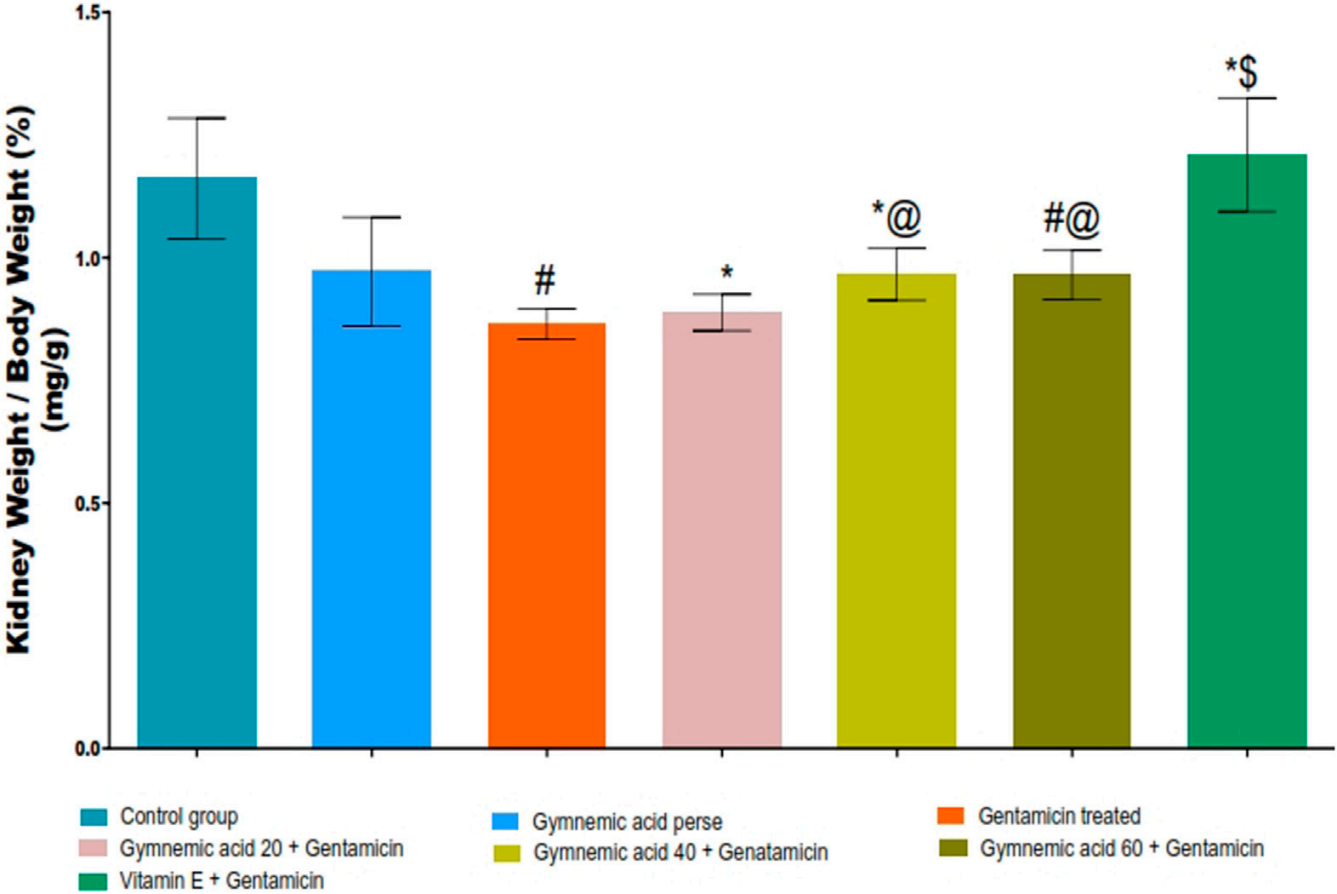
Effect of GA on kidney weight/body weight (%) in gentamicin-treated nephrotoxic rats. Data representing seven Groups per group of six rats in the above graph. Values were expressed as mean ± SD; Tukey’s multiple comparison tests analyzed data. ^#^ *p* < 0.001 versus vehicle control/GA 60; *p < 0.001 versus gentamicin treated; *@ *p* < 0.001 versus gentamicin and GA 20; ^#^@ *p* < 0.001 versus gentamicin and GA 40; *$ *p* < 0.001 versus gentamicin and GA 60.

The GEN control group rats had significantly (*p* < 0.01) lower kidney weight/body weight (%) than the control group. Oral administration of GA (20, 40, and 60 mg/kg, p.o., 2 weeks) to GEN-treated rats increased kidney weight/body weight (%) significantly (*p* < 0.001) and dose-dependently compared to GEN-treated control rats [F (6, 35) = 14.2]. In addition, vitamin E (8 mg/kg) significantly (*p* < 0.001) increased the percentage of kidney weight to body weight compared to GEN-treated control rats (Figure 4).

### 3.3 Effect of GA on mitochondrial-ETC complex I enzyme level in gentamicin-treated nephrotoxic rats

**FIGURE 5:**
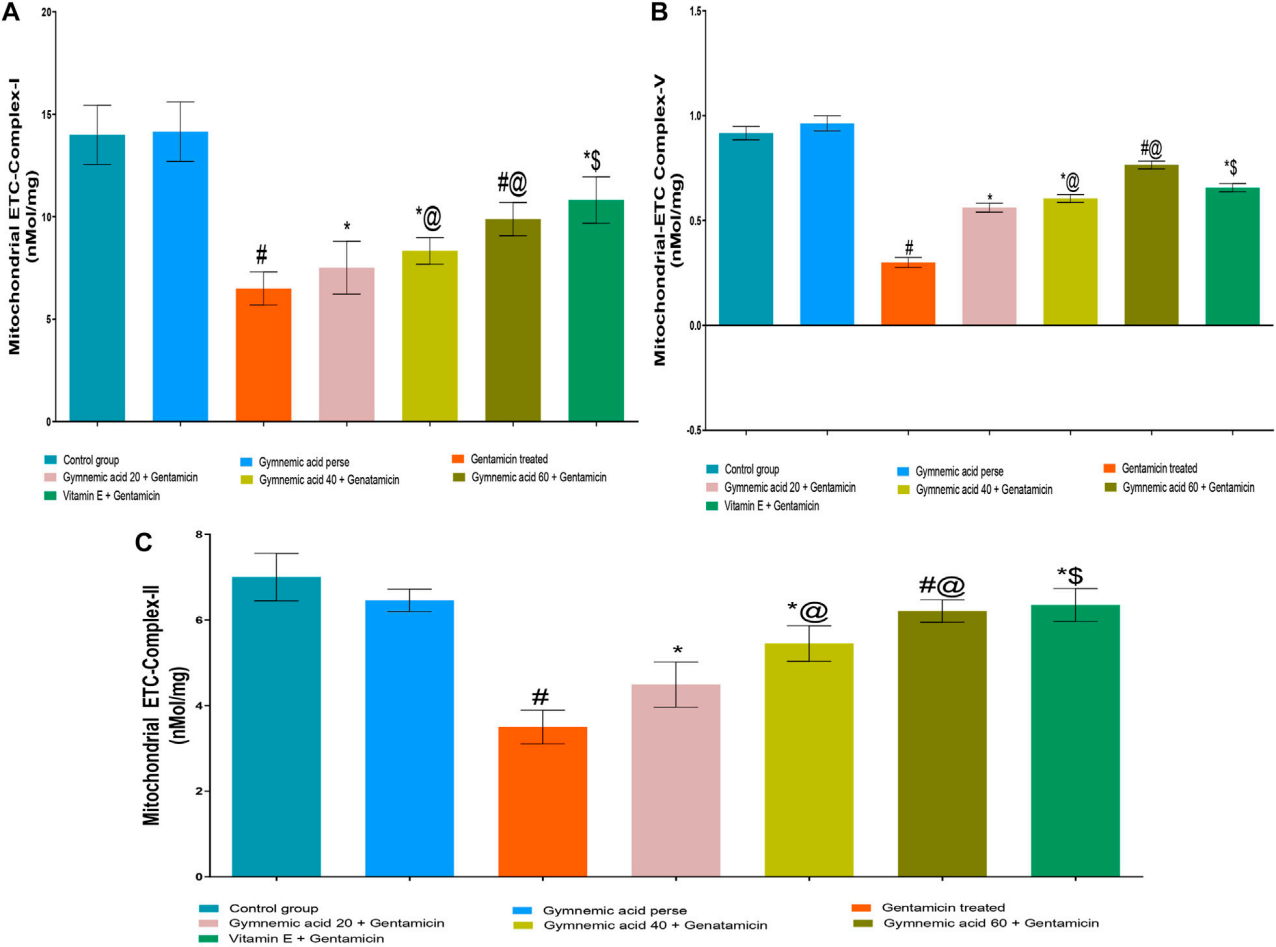
**(A–C)** Effect of GA on mitochondrial-ETC complex-I **(A)**; mitochondrial-ETC complex-V **(B)**; and mitochondrial-ETC complex-II **(C)** enzyme level ingentamicin-treated nephrotoxic rats. Data representing seven Groups per group of six rats in the above graph. Values were expressed as mean ± SD; Tukey’s multiple comparison tests analyzed data. ^#^ *p* < 0.001 versus vehicle control/GA 60; *p < 0.001 versus gentamicin treated; *@ *p* < 0.001 versus gentamicin and GA 20; ^#^@*p* < 0.001 versus gentamicin and GA 40; *$ *p* < 0.001 versus gentamicin and GA 60.

The mitochondrial-ETC complex-1 enzyme activity levels were significantly (*p* < 0.01) lower in GEN-treated rats than in controls. The oral administration of 60 mg/kg GA to rats treated with GEN increased the activity of the mitochondrial-ETC complex-1 enzyme. GA (20, 40, and 60 mg/kg, p.o., 2 weeks) administered to GEN-treated rats significantly and dose-dependently increased the mitochondrial-ETC complex-1 enzyme activity (*p* < 0.001) compared to GEN-treated control rats [F (5, 30) = 43.36]. In addition, vitamin E (8 mg/kg, p.o.) substantially increased the activity of the mitochondrial-ETC complex-1 enzyme in rats treated with GEN (Figure 5A).

### 3.4 Effect of GA on mitochondrial-ETC ATP synthase complex-V enzyme level in gentamicin-treated nephrotoxic rats

The mitochondrial-ETC ATP synthase complex-V enzyme activity levels in GEN-treated rats were significantly (*p* < 0.01) lower than in control rats. Compared to GEN-treated rodents, 60 mg/kg GA administration increased the mitochondrial-ETC ATP synthase complex-V enzyme activity. GA (20, 40, and 60 mg/kg, p.o., 2 weeks) administered to GEN-treated rats significantly and dose-dependently increased the mitochondrial-ETC complex-1 enzyme activity (*p* < 0.001) compared to GEN-treated control rats [F (5, 30) = 43.36]. Moreover, vitamin (8 mg/kg, p.o.) restored significantly (*p* < 0.001) the mitochondrial-ETC ATP synthase complex-V enzyme activity in GEN-treated rats (Figure 5B).

### 3.5 Effect of GA on mitochondrial-ETC complex II enzyme level in gentamicin-treated nephrotoxic rats

The mitochondrial-ETC complex-II enzyme activity levels in GEN-treated rats were significantly (*p* < 0.01) lower than in control rats. The administration of 60 mg/kg GA orally increased the activity of the mitochondrial-ETC complex-II enzyme in GEN-treated rats. GA (20, 40, and 60 mg/kg) administered to GEN-treated rats significantly and dose-dependently increased the mitochondrial-ETC complex-II enzyme activity (*p* < 0.001) compared to GEN-treated control rats [F (6, 35) = 54.17]. In addition, vitamin (8 mg/kg, p.o.) significantly (*p* < 0.001) increased the activity of the mitochondrial-ETC complex-II enzyme compared to rats treated with GEN (Figure 5C).

### 3.6 Effect of GA on MDA level in gentamicin-treated nephrotoxic rats

**FIGURE 6:**
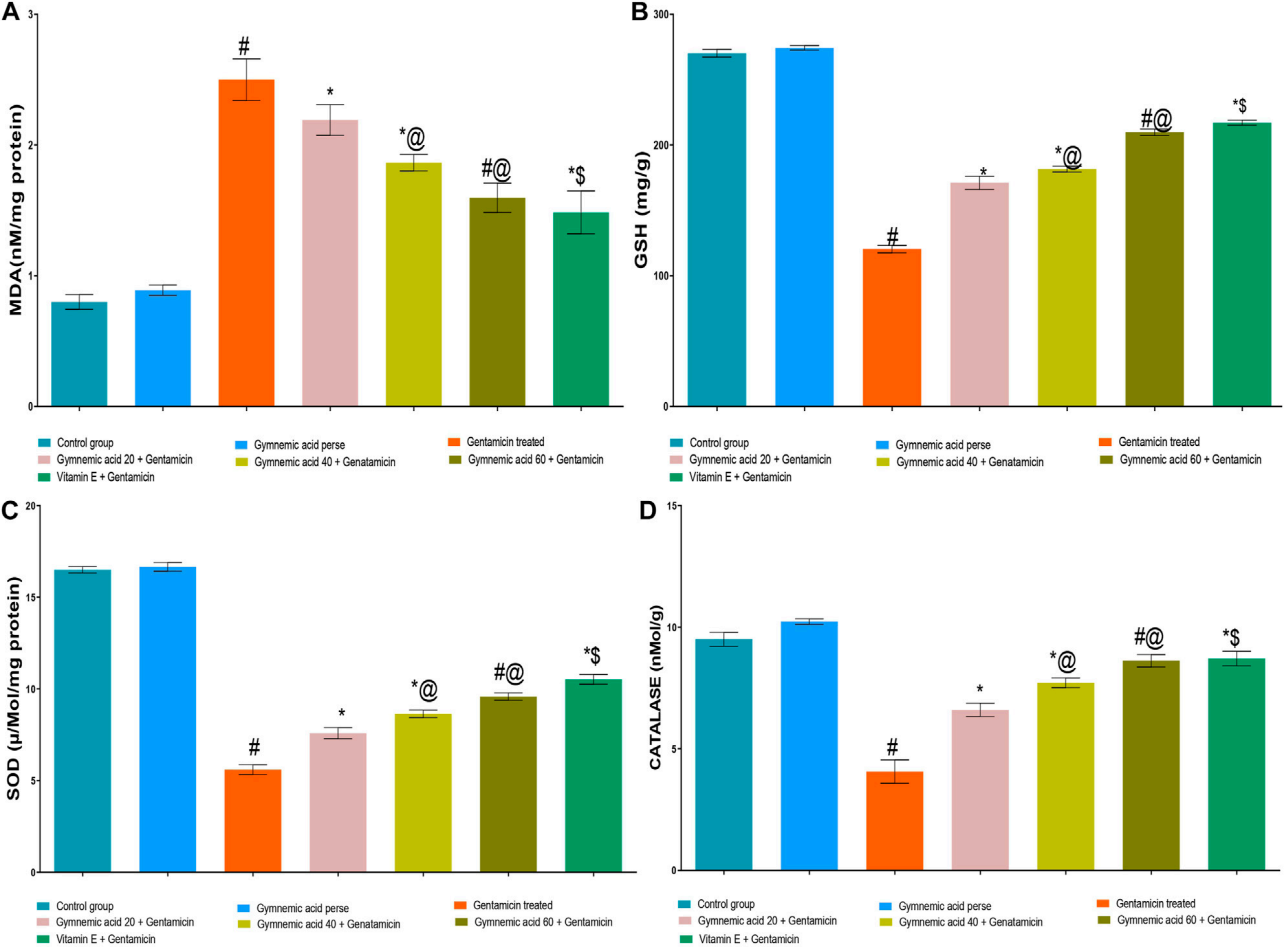
**(A–D)** Effect of GA acid on MDA **(A)**; glutathione **(B)**; SOD **(C)**; and catalase **(D)** levels in gentamicin-treated nephrotoxic rats. Data representing seven Groups per group of six rats in the above graph. Values were expressed as mean ± SD; Tukey’s multiple comparison tests analyzed data. ^#^ *p* < 0.001 versus vehicle control/GA 60; *p < 0.001 versus gentamicin treated l; *@ *p* < 0.001 versus gentamicin and GA 20; ^#^@*p* < 0.001 versus gentamicin and GA 40; *$ *p* < 0.001 versus gentamicin and GA 60.

GEN-treated rats had significantly (*p* < 0.01) higher MDA levels than normal rats. Orally administered GA at a dose of 60 mg/kg resulted in a lower MDA level in rats than GEN treatment. GA (20, 40, and 60 mg/kg, p.o., 2 weeks) administered to GEN-treated rats significantly and dose-dependently decreased the MDA level (*p* < 0.001) compared to GEN-treated control rats [F (6, 35) = 190.7]. In addition, vitamin E (8 mg/kg, p.o.) significantly (*p* < 0.001) decreased MDA levels in GEN-treated rodents (Figure 6A).

### 3.7 Effect of GA on GSH level in gentamicin-treated nephrotoxic rats

The levels of GSH in rats treated with GEN were significantly (*p* < 0.01) lower than those in normal rats. The administration of 60 mg/kg of GA orally increased GSH levels in rodents compared to those treated with GEN. GA (20, 40, and 60 mg/kg, p.o., 2 weeks) administered to GEN-treated rats significantly and dose-dependently increased GSH concentration (*p* < 0.001) in comparison to GEN-treated control rats [F (6, 35) = 2137]. Moreover, vitamin E (8 mg/kg, p.o.) significantly (*p* < 0.001) increased the GSH level in rats treated with GEN compared to rats treated with GEN alone (Figure 6B).

### 3.8 Effect of GA on SOD level in gentamicin-treated nephrotoxic rats

The GEN-treated rats had significantly (*p* < 0.01) lower SOD levels than the control rats. The administration of 60 mg/kg of GA orally significantly increased the SOD level. GA (20, 40, and 60 mg/kg, p.o., 2 weeks) administered to GEN-treated rats significantly and dose-dependently increased SOD (*p* < 0.001) in comparison to GEN-treated controls [F (6, 35) = 1879]. Moreover, vitamin E (8 mg/kg, p.o.) significantly (*p* < 0.001) increased the SOD level in rodents treated with GEN (Figure 6C).

### 3.9 Effect of GA on catalase level in gentamicin-treated nephrotoxic rats

Rats treated with GEN had significantly (*p* < 0.01) lower levels of catalase enzyme than control rats. Compared to rodents treated with GEN, oral administration of 60 mg/kg of GA increased catalase levels. The administration of 20, 40, and 60 mg/kg of GA to GEN-treated rodents increased catalase significantly and dose-dependently (*p* < 0.001) [F (6, 35) = 299.4]. In addition, vitamin E (8 mg/kg orally) significantly (*p* < 0.001) increased the SOD level in rodents treated with GEN (Figure 6D).

### 3.10 Effect of GA on inflammatory cytokines TNF-α level in gentamicin treated nephrotoxicity rats

**FIGURE 7:**
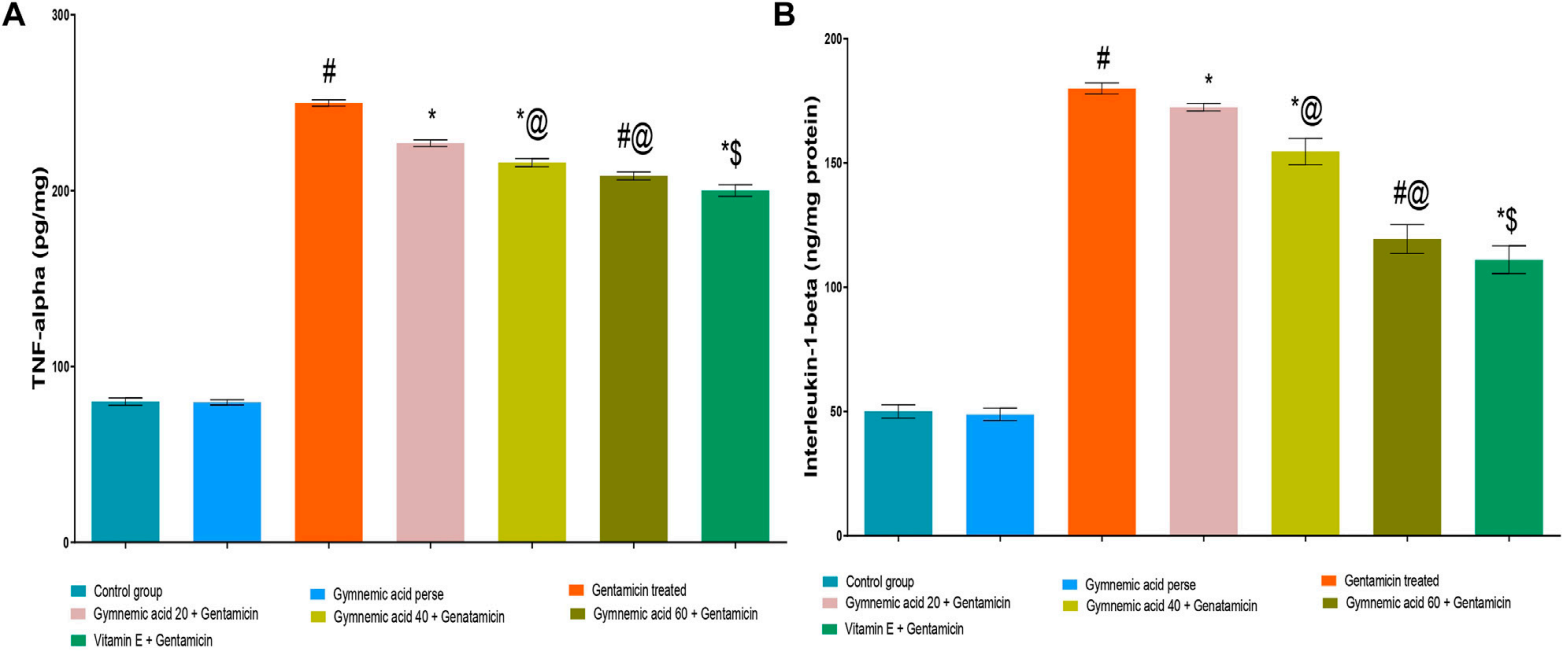
**(A,B)** Effect of GA on the TNF-α **(A)** and IL-1β **(B)** level in gentamicin-treated nephrotoxic rats. Data representing seven Groups per group of six rats in the above graph. Values were expressed as mean ± SD; Tukey’s multiple comparison tests analyzed data. ^#^ *p* < 0.001 versus vehicle control/GA 60; *p < 0.001versus gentamicin treated; *@ *p* < 0.001 versus gentamicin and GA 20; ^#^@*p* < 0.001 versus gentamicin and GA 40; *$ *p* < 0.001 versus gentamicin and GA 60.

Rats treated with GEN had significantly (*p* < 0.01) higher TNF-α cytokine levels than untreated rats. The oral administration of 60 mg/kg GA decreased TNF-α in rodents compared to those given GEN. The administration of GA at doses of 20, 40, and 60 mg/kg to GEN-treated rats significantly and dose-dependently decreased TNF-α (*p* < 0.001) compared to GEN-treated control rats [F (5, 30) = 7605]. In addition, compared to GEN-treated control rats, vitamin E (8 mg/kg., p.o.) significantly decreased TNF-α levels (*p* < 0.001) (Figure 7A).

### 3.11 Effect of GA on inflammatory cytokine IL-1β levels in gentamicin-treated nephrotoxicity rats

GEN-treated rats had (*p* < 0.01) increased levels of the cytokine IL-1β compared to untreated rats significantly. Rodents administered 60 mg/kg GA orally had lower levels of IL-1β than rodents administered GEN. GA (20, 40, and 60 mg/kg) decreased IL-1β in a dose-dependent manner (*p* < 0.001) in GEN-treated rats compared to GEN-treated control rats [F (6, 35) = 6277]. In addition, compared to GEN-treated control rats, vitamin E (8 mg/kg, p.o.) substantially decreased IL-1 levels (*p* < 0.001) (Figure 7B).

### 3.12 Effect of GA on gross and histopathological alterations in gentamicin-treated nephrotoxic rats

**FIGURE 8:**
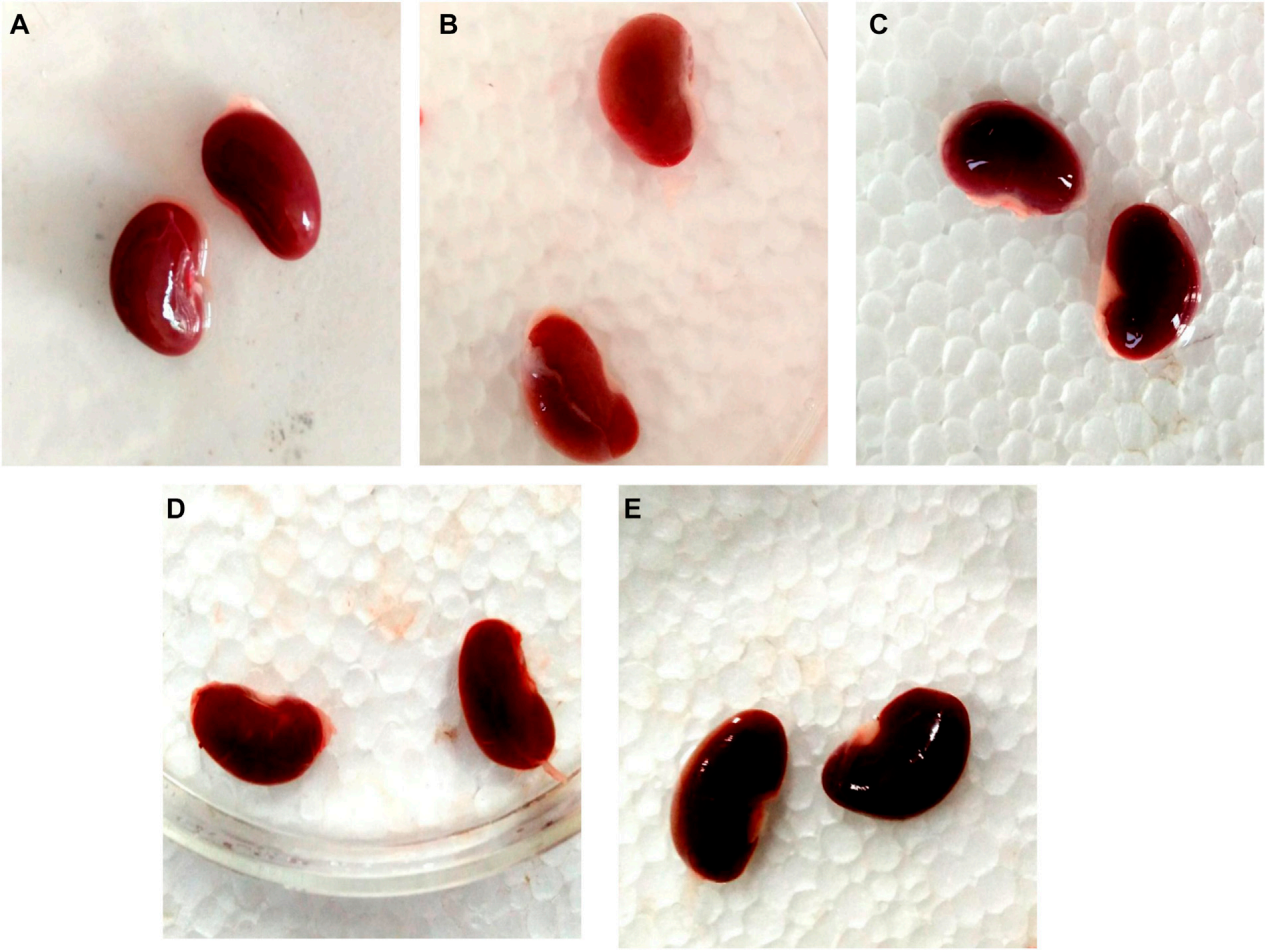
Nephroprotective potential of GA in the restoration of gross pathological changes in the kidney in gentamicin-treated rats; normal group renal tubules within normal limits, glomeruli within normal limits, no degenerative or atypical changes seen **(A)**; gentamicin treated group showing the severe tubular necrosis, glomerular mesangial hypercellularity, focal hemorrhage and chronic inflammation **(B)**; Renal tubules in vitamin E treated rats were within normal limits, glomeruli were within normal limits, and no degenerative or atypical changes were observed **(C)**. In the GA-20 treated group kidney showed tubular and glomerular degenerative changes (mild to moderate), degeneration is more in the renal cortex than the medulla **(D)**; in the GA -60 treated group kidney showed structure near to normal **(E)**.

**FIGURE 9:**
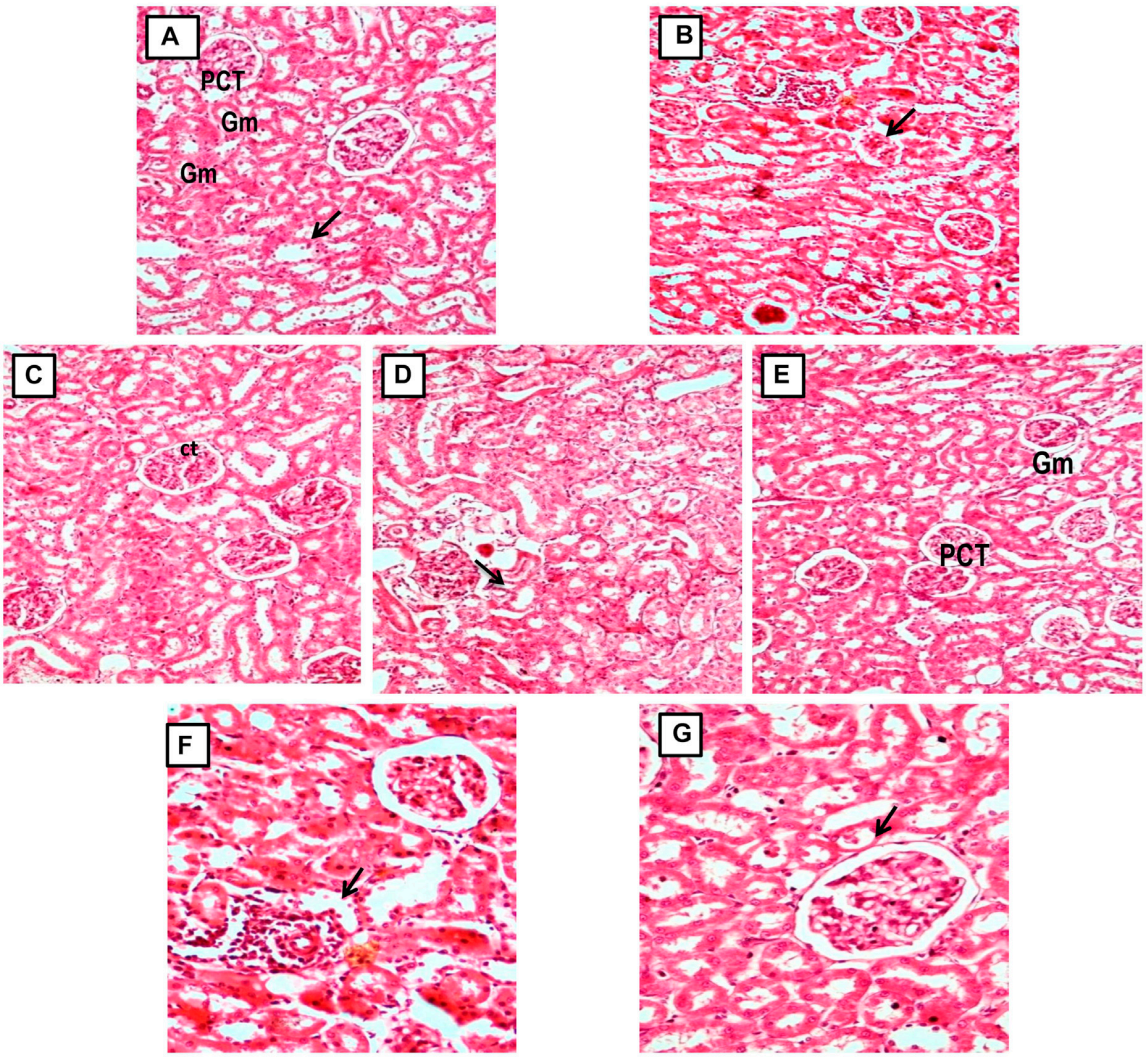
**(A, C)** Micrograph of group 1 and 3, Normal and GA 60 mg/kg Kidney group demonstrating normal histological structure of glomerulus (Gm) and renal tubules (PCT: Proximal convoluted tubules and CT: Collecting tubules); **(B)** Microscope image of kidney of gentamicin-treated group 2 demonstrating focal necrotic area with severe infiltration of lymphocytes (arrow) and shrunken glomeruli (Gentamicin 100 mg/kg, i.p; **(D, E)** Microscopy of Kidney from GA pretreated group 4 and 5 demonstrating restoration of degenerative changes in renal tubules (gentamicin 100 mg/kg, i.p. + GA 20 and 40 mg/kg); **(E, F)** Micrograph of group 6 and 7 showing reduction of kidney Tubular necrosis, glomerular mesangial hypercellularity, focal haemorrhage, and chronic inflammation (gentamicin 100 mg/kg, i.p. + GA 60 mg/kg and gentamicin 100 mg/kg, i.p. + Vitamin E 8 mg/kg).

In contrast to GEN-treated rats, GA-treated experimental animals exhibited dose-dependent restoration of body weight, GFR rate, serum creatinine, BUN, protein level, kidney weight/body weight (%), mitochondrial ETC complexes I, III, and V, and decreased levels of inflammatory cytokines, including TNF-α and IL-1β. In a dose-dependent manner, GA treatment decreased the renal oxidative stress marker MDA while increasing the levels of GSH, SOD, and catalase. Compared to GA, vitamin E played a significant role in restoring all of these biochemical markers. Histological examinations confirmed the renoprotective properties of GA (Figures 8 and 9).

## 4 Discussion

This investigation has unveiled an associative relationship between acute renal failure and the consumption of antibiotics, predominantly aminoglycosides. These broad-spectrum antibiotics have been significantly implicated in numerous studies, characterized by aminoglycoside-induced nephrotoxicity in laboratory animals (Krause et al., 2016; Laorodphun et al., 2022). The present research was designed to elucidate whether GA could provide a defensive mechanism against gentamicin-induced renal impairment.

Gentamicin treatment has been found to precipitate apoptosis and necrotic degeneration of tubular epithelial cells in lab rats, primarily via the modulation of phospholipid metabolism (Laurent et al., 1983; El Mouedden et al., 2000; Edwards et al., 2007; Li et al., 2009). Additional factors potentially exacerbating or predisposing the condition, such as regional renal ischemia, might also impact its progression. Standard measurements of nephrotoxicity frequently comprise serum creatinine, blood urea, urea protein content, GFR, and other renal function indices (Basile et al., 2012; Makris and Spanou, 2016).

Pro-inflammatory cytokines, particularly IL-1β, produced by inflammatory cells, tend to increase during renal disease. IL-1β, an essential component of the host’s defense mechanism against infection and injury, is known to play a pivotal role in regulating the inflammatory response (Zhang and An, 2007; Tbahriti et al., 2013; Dinarello, 2018). This cytokine, one of the most extensively studied members of the IL-1β family, can be produced and secreted by various cell types, primarily monocytes and macrophages of the innate immune system (Dinarello, 2018). Pro- IL-1β, an inactive cytokine precursor with a molecular weight of 31 kilodaltons, is induced by pathogen-associated molecular patterns (PAMPs) acting on the pattern recognition receptors (PRRs) of macrophages (Takeuchi and Akira, 2010; Lopez-Castejon and Brough, 2011).

Traditional medicine globally relies on more natural approaches to prevent and manage renal problems, albeit their usage, side effects, and clinical assessment remain underexplored. Dietary consumption of natural antioxidants may help mitigate cellular damage (Boaz et al., 2000; Ceriello, 2003; Stanifer et al., 2015; Yang et al., 2018). *G. sylvestre* leaf, a traditional Ayurvedic remedy, has been used for centuries for its anti-inflammatory, astringent, acrid, thermogenic, digestive, and hepatoprotective properties. It is also utilized in naturopathy to treat a variety of conditions, including diabetes, obesity, arthritis, hyperlipidemia, parkinson’s disease, and hypercholesterolemia (Tiwari et al., 2014; Yang et al., 2018). Decreasing necrosis and other cellular aberrations could potentially mitigate nephrotoxicity resulting from aminoglycosides. Previous studies have identified antioxidant capabilities within GA (Tiwari et al., 2014; Khan et al., 2019; Jangam et al., 2023).

The standard drug, vitamin E, significantly ameliorated these renal function biomarkers. Parallel studies by El-Ashker et al. (2015) in Egypt identified lower levels of IL-1β, interferon-gamma (IFN-γ), MDA, and serum creatinine in the vitamin C group than in the control group. Similarly, GA (60 mg/kg) remedied the elevated levels of inflammatory cytokines like TNF-α and IL-1β induced by gentamicin (Lopez-castejon and Brough, 2011). An analogous study in Thailand reported that gentamicin-induced renal injury led to increased oxidative and endoplasmic reticulum (ER) stress. The antioxidant curcumin (200 mg/kg/day) mitigated renal oxidative stress markers such as BUN, MDA, GSH, SOD, and serum creatinine (Laorodphun et al., 2022).

In the present study, the gentamicin-treated group witnessed a considerable decrement in kidney weight, whereas the GA-treated group saw a slightly less substantial reduction. We compared the urinary volume, serum creatinine, and GFR of each group with the controls. GA demonstrated remarkable efficiency in restoring antioxidant potential and mitochondrial electron transport chain (ETC) complex enzymes, exceeding that of Vitamin-E in the gentamicin, GA, and Vitamin-E-treated groups. GA treatment effectively restored renal oxidative stress markers, including MDA, GSH, SOD, BUN, and serum creatinine. These biomarkers showed significant improvement with escalating GA doses of 20, 40, and 60 mg/kg.

In another study, histopathological examination demonstrated that gentamicin treatment prompted coagulative necrosis in the tubular epithelial membrane of the renal cortex, leading to the infiltration of inflammatory cells among degenerated tubules. However, diosmin (100 mg/kg) prompted minimal inflammatory cell infiltration into the renal tubule cortex. The anti-inflammatory compound diosmin reduced the count of TNF-α, IL-1β, p38 mitogen-activated protein kinase (p38MAPK), and nuclear factor kappa B (NF-ĸB) cells (Nadeem et al., 2023). Consistent with the current findings, Haghighi et al. (2012) reported a decrease in animal body weight following nephrotoxicity. Changes in normalized kidney weight may be a reliable predictor of renal pathology (Basile et al., 2012; Haghighi et al., 2012).

Histopathological analysis revealed morphological changes within the kidney that provide additional dimensions for organ evaluation. In the gentamicin-treated group, renal histology showed substantial inflammatory cell infiltration and renal tubular damage, along with significant degeneration of the tubule lining epithelium, vascular congestion, tubular edema, and a focal necrotic area with minor lymphocytic infiltration and reduced glomeruli. GA pretreatment resulted in a reduced extent of these morphological alterations. The 40 mg/kg GA group displayed moderate degenerative changes and cellular edema in renal tubules. Meanwhile, the 60 mg/kg GA dose significantly mitigated the injury severity in the glomerulus, renal tubules, proximal convoluted tubules, and collecting tubules (Figures 8 and 9).

## 5 Limitations

These results suggest that GA may possess therapeutic potential against GEN-induced nephrotoxicity; however, its efficacy in treating acute renal failure warrants further exploration. Subsequent research efforts should focus on the molecular mechanism of GA action that may incorporate techniques like, immunohistochemistry, western blotting, and gene expression studies.

## 6 Conclusion

The current study assessed the preventative potential of GA against gentamicin-induced renal dysfunction, commonly marked by inflammation and cell necrosis. Gentamicin promotes cell apoptosis and necrosis in kidney tubular epithelial cells by disrupting phospholipid metabolism. This antibiotic-induced damage was measured by indicators such as serum creatinine, blood urea, protein in the urine, and GFR. Gentamicin incites mitochondrial ETC complex dysfunction, and inflammation, releasing pro-inflammatory cytokines like IL-1β, a vital component of the host’s defense against infection and injury. However, the treatment with GA significantly improved kidney health markers such as MDA, GSH, SOD, BUN, and serum creatinine at varying doses. It was also more effective than Vitamin E in restoring antioxidant potential and mitochondrial-ETC complex enzymes. The protective effects of GA was further confirmed by histopathological examinations. In addition, the study observed decreased animal body weight following nephrotoxicity, highlighting the correlation between normalized kidney weight changes and renal pathology. In gentamicin-treated groups, significant renal damage was observed, including inflammatory cell infiltration, tubular injury, and necrosis. However, pre-treatment with GA reduced these symptoms, with a 60 mg/kg dose significantly decreasing the severity of kidney injury. In comparison to similar studies, GA’s ability to reduce inflammation and oxidative stress was akin to the effects of antioxidants such as vitamin C. This demonstrates the potential of GA in mitigating drug-induced kidney damage.

## Data Availability

The original contributions presented in the study are included in the article/Supplementary Material, further inquiries can be directed to the corresponding authors.

## Abbreviations

ACE, Angiotensin-converting enzyme inhibitor; ARB, Angiotensin receptor blocker; CPCSEA, Committee for the Purpose of Control and Supervision of Experiments on Animals; CT, Collecting ducts; GFR, Glomerular filtration rate; GM, glomerulus; GEN, Gentamicin; GA, Gymnemic acid; NSAID, Nonsteroidal anti-inflammatory drugs; PG, Prostaglandins; PCT, Proximal convoluted tubules; ROS, Reactive oxygen species; SEM, Standard error of the mean; UF, Ultrafiltration.
